# Asymmetrical Damage Partitioning in Bacteria: A Model for the Evolution of Stochasticity, Determinism, and Genetic Assimilation

**DOI:** 10.1371/journal.pcbi.1004700

**Published:** 2016-01-13

**Authors:** Lin Chao, Camilla Ulla Rang, Audrey Menegaz Proenca, Jasper Ubirajara Chao

**Affiliations:** 1 Section of Ecology, Behavior and Evolution, Division of Biological Sciences, University of California, San Diego, La Jolla, California, United States of America; 2 CAPES Foundation, Ministry of Education of Brazil, Brasilia, Brazil; University of New South Wales, AUSTRALIA

## Abstract

Non-genetic phenotypic variation is common in biological organisms. The variation is potentially beneficial if the environment is changing. If the benefit is large, selection can favor the evolution of genetic assimilation, the process by which the expression of a trait is transferred from environmental to genetic control. Genetic assimilation is an important evolutionary transition, but it is poorly understood because the fitness costs and benefits of variation are often unknown. Here we show that the partitioning of damage by a mother bacterium to its two daughters can evolve through genetic assimilation. Bacterial phenotypes are also highly variable. Because gene-regulating elements can have low copy numbers, the variation is attributed to stochastic sampling. Extant *Escherichia coli* partition asymmetrically and deterministically more damage to the old daughter, the one receiving the mother’s old pole. By modeling *in silico* damage partitioning in a population, we show that deterministic asymmetry is advantageous because it increases fitness variance and hence the efficiency of natural selection. However, we find that symmetrical but stochastic partitioning can be similarly beneficial. To examine why bacteria evolved deterministic asymmetry, we modeled the effect of damage anchored to the mother’s old pole. While anchored damage strengthens selection for asymmetry by creating additional fitness variance, it has the opposite effect on symmetry. The difference results because anchored damage reinforces the polarization of partitioning in asymmetric bacteria. In symmetric bacteria, it dilutes the polarization. Thus, stochasticity alone may have protected early bacteria from damage, but deterministic asymmetry has evolved to be equally important in extant bacteria. We estimate that 47% of damage partitioning is deterministic in *E*. *coli*. We suggest that the evolution of deterministic asymmetry from stochasticity offers an example of Waddington’s genetic assimilation. Our model is able to quantify the evolution of the assimilation because it characterizes the fitness consequences of variation.

## Author Summary

The benefit of non-genetic variation in phenotypic traits is debated. We show that the partitioning of damage by a mother bacterium to its two daughters is a variable trait that provides an advantage by generating fitness variation. Present day bacteria partition asymmetrically more damage to the old daughter, the one receiving the mother’s old pole. By modeling damage partitioning in a population, we find that the asymmetry is advantageous because it increases fitness variation and hence the efficiency of natural selection. However, our model also shows that symmetrical but randomly variable partitioning can be similarly beneficial. To examine why bacteria evolved asymmetry, we modeled the effect of damage anchored to the mother’s old pole. While anchored damage strengthens selection for asymmetry by creating additional fitness variation, it has the opposite effect on symmetry. Thus, symmetrical but randomly variable partitioning may have been sufficient to protect early bacteria from damage. However, natural selection then supplanted it with a genetically controlled mechanism in the form of asymmetrical partitioning. The change from random to genetic control is an important evolutionary transition that is poorly characterized. We are able to quantify this transition because our model estimates the costs and benefits of fitness variation.

## Introduction

The costs and benefits of non-genetic phenotypic variation are a long-standing topic of interest in evolutionary biology [1–3]. While the variation introduces a cost by generating suboptimal phenotypes that fail in a set of environments, it also provides a benefit by generating plastic phenotypes that may better match changing or new conditions. The variation is non-genetic because it results from stochasticity or noise in the expression of genes or developmental pathways controlling the phenotype. The chance matching to change corresponds to a bet-hedging strategy. However, if the new conditions become long term, natural selection shifts to favor genetic modifications in which the initial trait evolves from being stochastically determined to become genetically controlled.

The transition from stochasticity to genetic determinism was independently suggested by Waddington and Schmalhausen [4–6], who also introduced the terms genetic assimilation to denote the process, and canalization to represent the trait’s increasing robustness, or ability to resist environmental perturbations, during assimilation. Working with *Drosophila melanogaster*, Waddington was motivated by his observations on the evolution of the crossveinless (CVL) phenotype, which appeared as a gap in the venation of the fly wing. The phenotype was initially determined by environmental factors because wild type flies, which were not CVL under normal conditions, expressed the gap after their pupae were exposed to a brief heat shock. Waddington then selected for the CVL phenotype after heat shock by using flies expressing the gap as the parents for the next generation. After several generations, not only did the frequency of CVL flies increase in the population, but the flies also had evolved to express the gap without a heat shock. Thus, the CVL phenotype had become genetically assimilated.

**Fig 1:**
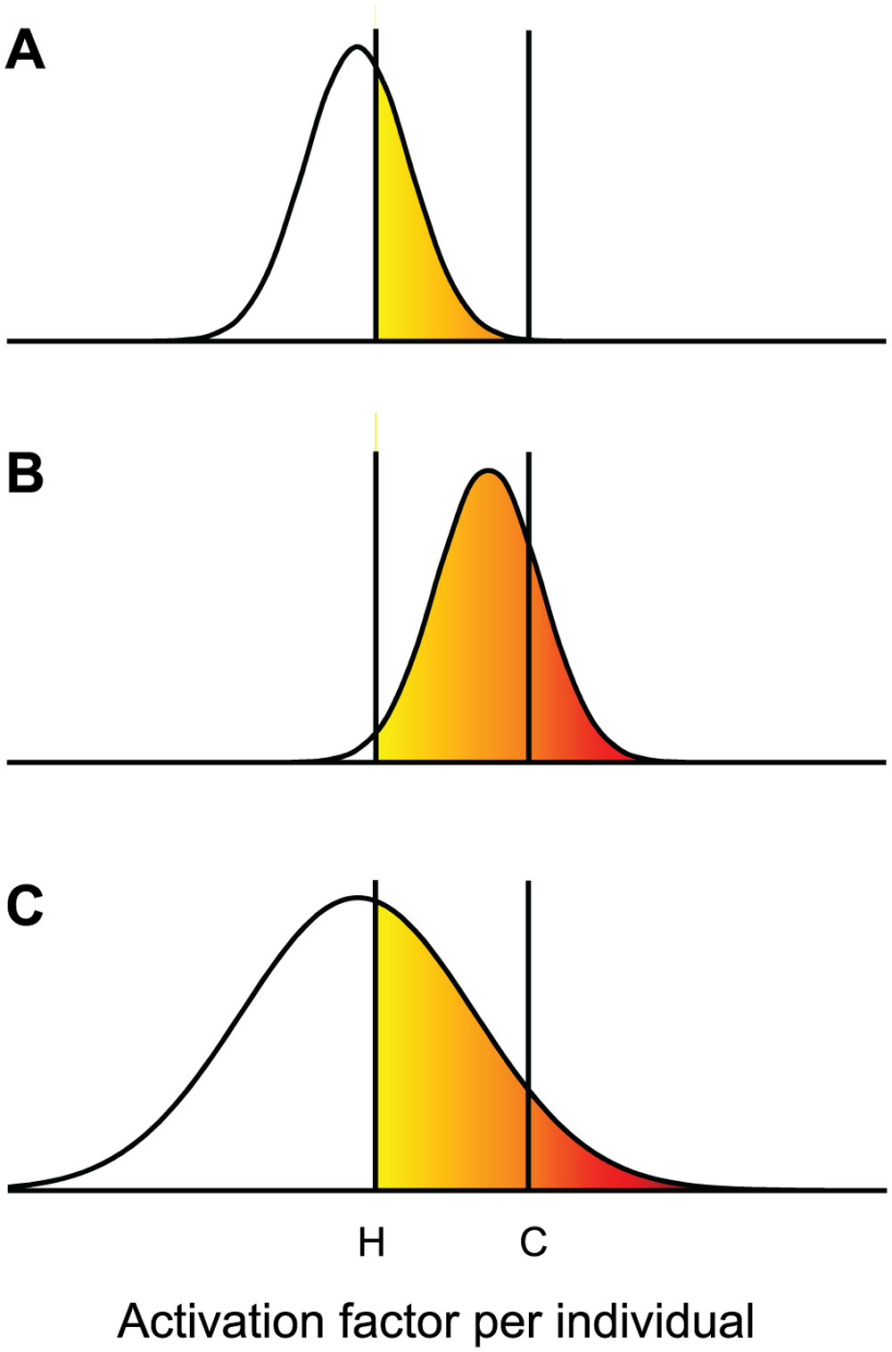
Evolution of genetic assimilation. An activation factor is assumed to be needed to express a phenotype such as crossveinless (CVL). The factor is produced stochastically and its concentration varies between individuals within a population. For CVL to be expressed the concentration needs to exceed a threshold. (A) Under Control conditions the threshold has a value C, and CVL is not expressed because no fly in a pre-selection wild type population exceeds the threshold. The effect of subjecting a pre-selection fly pupa to a heat shock is to lower the threshold to a value H, in which case some flies become CVL (yellow fraction). (B) After selection for CVL following heat shock, the selected flies evolved to produce the activation factor with a distribution that has a higher mean. Under Control conditions, more selected flies are CVL after heat shock (yellow and red fraction), but some flies are able to express CVL even under Control conditions (red fraction). (C) Alternatively, selected flies may have evolved an activation factor with a distribution that has a larger variance but the same mean as before selection. CVL is expressed under both Control conditions (red) and after heat shock (yellow and red).

The evolution of Waddington’s genetic assimilation can be explained by invoking an activation process (Fig 1). In order for CVL to be expressed, an individual fly must produce an activation factor above a threshold. However, the factor is produced with a stochastic distribution. In wild type flies before selection, the upper tail of the distribution is below the control threshold and CVL is not expressed. The effect of the heat shock is to lower the threshold so that some flies can now express CVL. Waddington’s selection evolved flies that expressed CVL at a higher frequency after heat shock. If the higher frequency was achieved by evolving a new distribution that was shifted upwards, some of the selected flies would now exceed the threshold even in the absence of heat shock. An alternative mechanism that increases the variance instead of the mean of the distribution could also explain the expression of CVL in the selected flies (Fig 1C). In the latter case, genetic assimilation would have required the evolution of a deterministic process to increase the variance above the original stochastic level.

To date, most studies examining the evolution of canalization and genetic assimilation have focused on metazoan examples [7]. This pattern is not surprising given that estimates of phenotypic variation require measurements of individuals within a population. Because of their small size, microbes, bacteria, fungi, and other single-celled organisms, have traditionally been studied mainly through the mean properties of their populations. However, with the recent advances in the resolution of optical microscopy and, most importantly, computer assisted automated time-lapse photo-microscopy, quantifying the phenotype of individual cells has become much easier. Elongation, division and growth rates can be obtained from time-lapse images [8–12], while gene expression within single-cells can be quantified by the use of fluorescence protein reporters [13–16]. The studies generally report a high level of non-genetic phenotypic variation between individuals within a population of genetically identical cells. By fitting a binomial distribution to the difference between the gene expression levels of two daughter cells descending from the same mother bacterium, the copy number of gene-regulating elements, e.g. a repressor protein, has been estimated to be small [13]. Thus, much of the variation in gene expression may be explained by stochastic or random sampling of the regulatory elements. Ensuing discussions raised again the need to assess the evolutionary consequences of phenotypic stochasticity, but now at the level of single cells [2, 17–19]. Issues similar to those discussed by Waddington and Schmalhausen were noted. In particular, what were the benefits and costs of non-genetic phenotypic variation at the fitness level?

The possibility of using microbes to model and test the evolution of canalization and genetic assimilation is appealing. Their ease of culture, short generation times, and amenability to genetic manipulation would allow for using experimental evolution to study the process in real time by natural selection. Although Waddington observed evolutionary changes, he used artificial selection and the fitness benefit of crossveined is unknown. Additionally, because many mathematical and computational models have been developed to describe specifically the generation of phenotypic variation in bacteria [20–24], the experiments can be designed and analyzed in coordination with theory.

Here we present a computational study aimed at identifying the potential costs and benefits of non-genetic stochastic variation in the growth rate phenotype of bacterial cells. We employ a model that we first developed to describe the process by which a mother bacterium deterministically partitions in an asymmetric manner her load of non-genetic damage, for example oxidized macromolecules, to her two daughters [20]. We chose this model because it was designed to complement bacterial data. All of its parameters and several of its key predictions have been estimated or tested with experimental studies [25, 26]. The model has revealed that asymmetrical partitioning is advantageous because it increases the fitness of a lineage by generating phenotypic variation and improving the efficiency of natural selection [20]. A more intuitive explanation is provided by the analogy of two compounded interest accounts, one starting with $100 at 8% yr^-1^ and a second split accounts that starts with $50 at 6% and $50 at 10%. Over time, the split accounts will accrue more money. Asymmetrical partitioning splits the lineages, the lineages with less damage correspond to the 10% account, and exponential growth by binary fission provides the compounded interest. The relationship between fitness variation and the efficiency of natural selection was first recognized by Fisher, who named it the Fundamental Theorem of Natural Selection [27]. However, although our model has helped our understanding of the evolutionary advantage of asymmetrical partitioning as a deterministic process, the effect of stochastic variation on damage partitioning in the model has previously not been investigated.

Our results show that stochastic partitioning, in the absence of asymmetrical partitioning, also provides a fitness advantage, but asymmetrical partitioning is more advantageous when some damage is anchored to the older poles of the daughter cells. We propose that asymmetrical partitioning, the extant phenotype in bacteria that have been examined [9, 10], evolved over stochastic partitioning because the latter is unable to match the polarity of the anchored damage. Asymmetrical partitioning matches that polarity better because it directs deterministically non-anchored damage to the older pole. By matching polarity, deterministic asymmetry increases even further the variance of damage partitioning. We suggest that the evolution of deterministic asymmetry over stochastic partitioning is an example of genetic assimilation. The only difference between Waddington’s results and ours is that the variation of the trait, rather than the trait itself, is assimilated. We hope that future experiments, both with microbes and metazoans, are motivated by these results.

## Results

### Basic model of damage partitioning in bacteria

We present first our basic model of damage partitioning, which will be later modified to incorporate stochastic partitioning. We provide here only an abbreviated summary, which should be sufficient for understanding the application of the model. A more detailed derivation and analysis is provided in Methods and the original publication [20]. We note that the equations presented here will not be numbered sequentially because some steps presented in Methods are omitted. The equations retain the numbering as in Methods for consistency.

**Fig 2:**
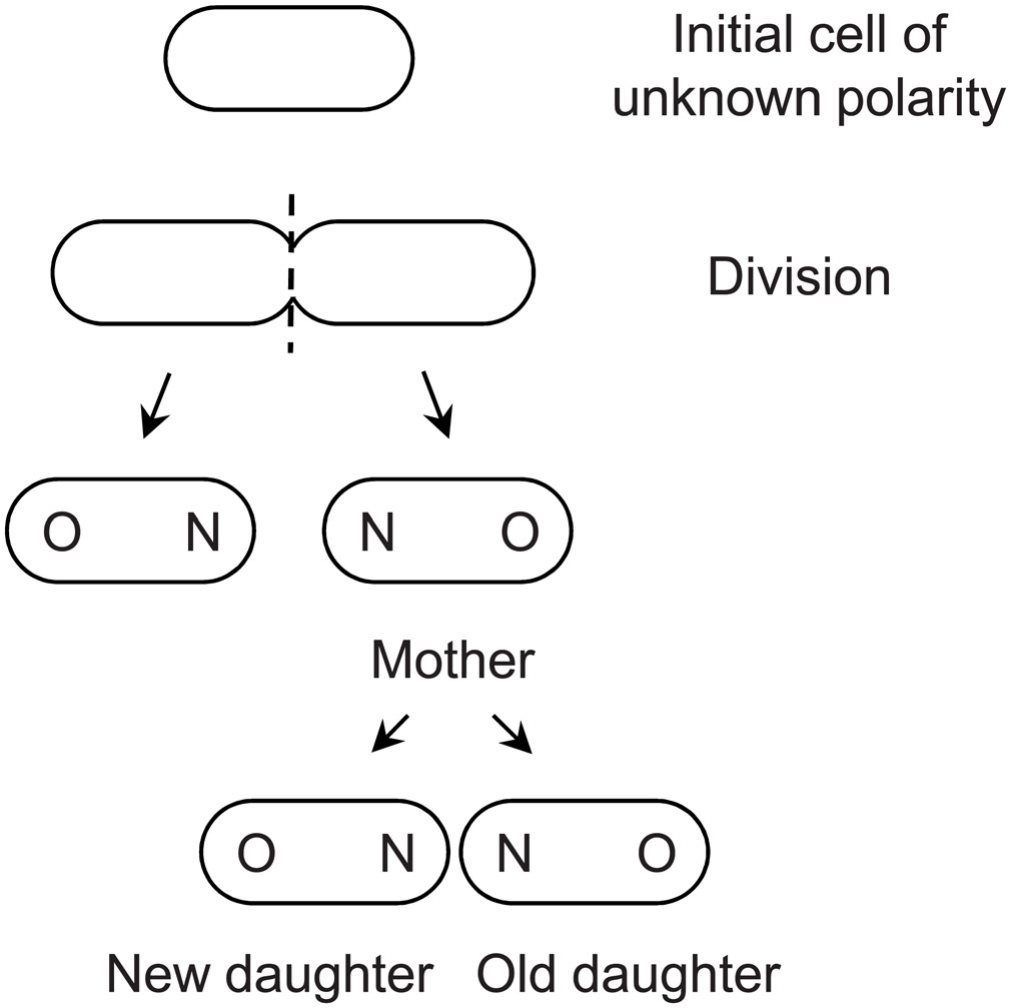
Cell polarity in *E*. *coli* cells. The cell polarity can be determined by tracking a lineage. Because division cleaves the short axis (-—-) of the cell, poles formed at the cleavage are new (N) and distal poles are old (O). After the next division, the daughter receiving the mother’s new pole is the new daughter and the other is the old daughter.

The basic model is built on the result that *E*. *coli* cell division is deterministically asymmetric because a mother cell allocates more non-genetic damage to her old daughter [9, 12]. The old and new daughter notation results from the division of rod-shaped bacteria such as *E*. *coli* when the septum cleaves the long axis of the cell (Fig 2). Because two new poles are formed at the septum, poles distal to the septum are the old poles. All bacteria, including mother and daughter bacteria, have a new and an old pole. Whenever a mother bacterium divides, one daughter receives the maternal old pole and the other receives the maternal new pole. The former is denoted the old daughter and the latter the new daughter.

The model has three parameters, *a*, *λ*, and *Π*, which are the asymmetry coefficient, the damage rate constant, and the doubling time of the fittest cell with no damage. The coefficient *a* measures the amount of damage a mother partitions to the new daughter and it has a value range of 0 ≤ *a* ≤ ½. A value of *a* = ½ denotes symmetrical partitioning. The doubling time is the number of minutes required for a bacterial cell to elongate and divide into two daughters. *Π* represents therefore the shortest doubling time possible for the bacteria.

A bacterium with damage has a doubling time *T* that is greater than *Π*. Our basic model derives its doubling time to be

$$T_{i}=\left\{\left(1–k_{i}\right)–\surd \left({\left(1–k_{i}\right)}^{2}–2\Pi \lambda \right)\right\}/\lambda$$ (7)

where *i* has a value of either 1 or 2 to denote the doubling time of either a new or old daughter, respectively, *k*_*i*_ is the amount of damage a mother cell partitions to the daughters when it divides, and

$$k_{1}=\;(k_{0}+\;\lambda T_{0})a$$ (4)

$$k_{2}=\;(k_{0}+\;\lambda T_{0})(1\;–\;a)$$ (5)

With asymmetrical partitioning, *k*_*1*_ *< k*_*2*_ because *a* < ½. *T*_*0*_ is the doubling time of the mother and *k*_*0*_, the amount of damage it got from its mother, is given as

$$k_{0}=\;1\;–\;\left(\lambda \;/\;2\right)\;T_{0}–\;\Pi \;/\;T_{0}$$ (8)

Thus, the amount of damage a mother partitions to her daughters is *k*_*0*_ plus the amount of new damage it accumulates over its lifetime, which equals *λT*_*0*_.

The power of the model is that if the doubling time *T*_*0*_ of the mother is known, the doubling times *T*_*1*_ and *T*_*2*_ can be predicted.

### Model for stochastic partitioning

To examine the effect of stochastic partitioning, we first modified our basic model by allowing the values of *a* to vary randomly. While everything else was left unchanged, each time that a cell reproduced in the population, its daughters were generated by Eqs 4 and 5 with a single value that was sampled from a Gaussian distribution with a mean *a* and a variance σ_S_^2^.

**Fig 3:**
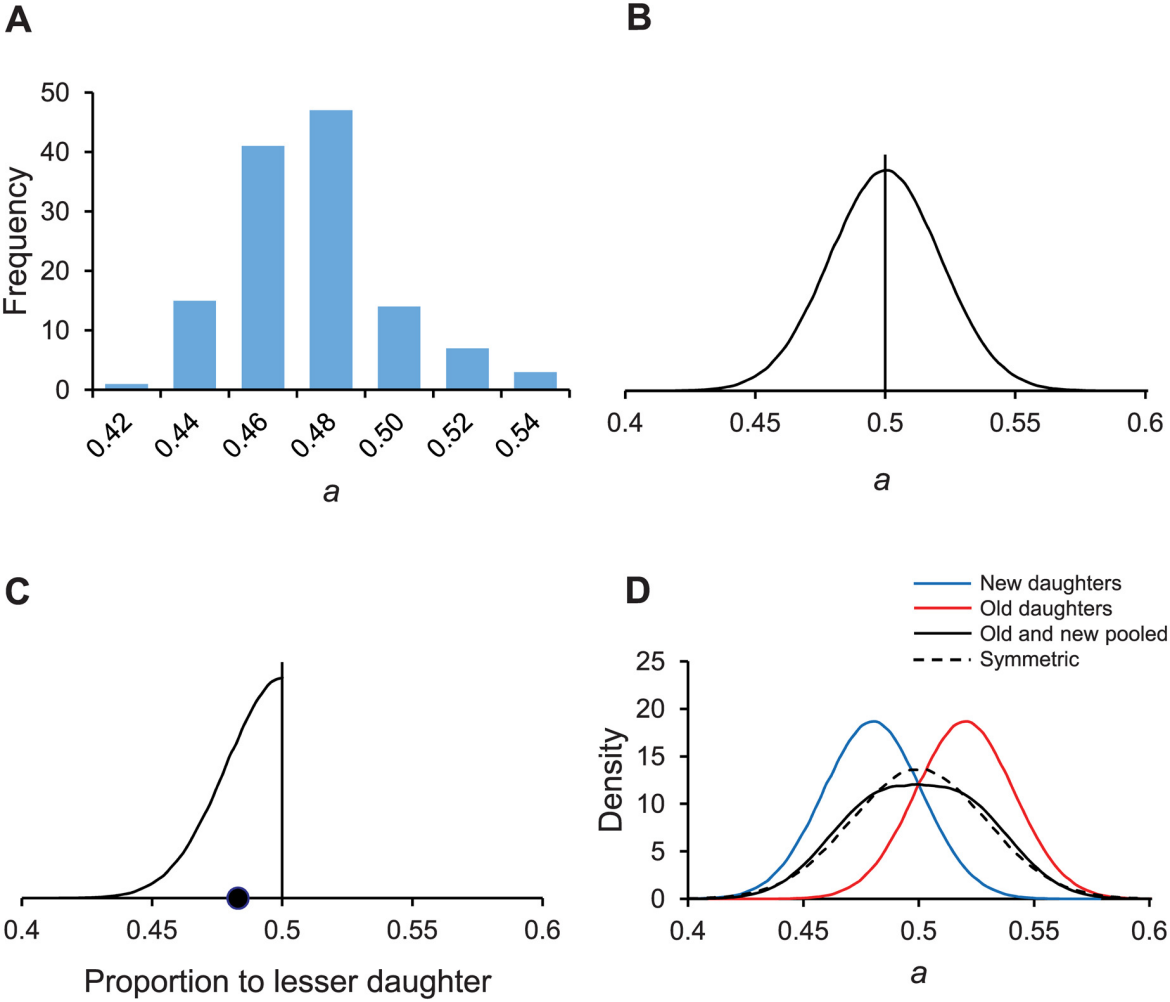
Distributions of the asymmetry coefficient *a*. The value of *a* represents the proportion of damage partitioned by a mother bacterium to its new daughter. Asymmetry requires that *a* < ½. If *a* = ½, the partitioning is symmetrical. Distributions are illustrative representations except for (A), which was derived from the experiments of Stewart et al. [9]. (A) Stochastic variation for observed values of *a* estimated from experimental *E*. *coli* data. Distribution mean = .4845, variance σ_S_^2^ = .0004557, and sample size n = 128. (B) Distribution of *a* when the partitioning of damage is stochastic but symmetrical with a mean of ½. A Gaussian distribution with a variance of σ_S_^2^ = .0004557 is assumed for illustration. (C) Distribution of the proportion of damage allocated to the daughter that gets less damage when partitioning is stochastic but symmetrical. Because symmetrical partitioning is random with respect to whether a daughter is old or new, polarity can be ignored and all the daughters can be re-categorized into ones that get less and ones that get more damage. If only the lesser daughters are considered, the resulting distribution is the half- or folded normal of the Fig 3B distribution. The mean of the half-normal is ½—√(σ_S_^2^ • 2 / 3.141593…), which equals .483 (●). (D) Gaussian distributions representing four populations: *a* of new daughters (mean = .48; var = σ_S_^2^ = .00046; *a* of old daughters (mean = 1 –.48 = .52; var = σ_S_^2^ = .00046); a population made by pooling the new and old daughters; and daughters produced by a stochastic but symmetric mother where the variance is increased to σ_S_^2^ + *D* ^2^/4 = .00046 + .0004^2^/4 = .00086 and mean = ½.

To obtain values of *a* and σ_S_^2^, we reevaluated the data of Stewart et al. [9], which we had previously used to estimate the parameters *a*, *λ*, and *Π* [20]. The data consisted of 128 trios of observed values of *T*_*0*_, *T*_*1*_ and *T*_*2*_. For each trio, the *T*_*1*_ and *T*_*2*_ corresponded to the actual daughters produced by the *T*_*0*_ mother. Our estimates were obtained by taking the observed values of *T*_*0*_, using our basic model to obtain predicted values of *T*_*1*_ and *T*_*2*_, and then finding parameter values that minimized the difference (squared deviation) between the predicted and observed *T*_*1*_ and *T*_*2*_. Those estimates were only for parameter means, which were *a* = .4843, *λ* = 0.0077 min^-1^, and *Π* = 18.95 min, because the 128 differences were pooled and minimized as one number. To estimate σ_S_^2^, we reanalyzed the data set by fitting our model to each individual trio, minimizing the difference, and obtaining 128 separate estimates of *a*, *λ*, and *Π*. From the mean and variances of the 128 estimates (Fig 3A), *a* = .4845 and σ_S_^2^ = .000456. Mean values for the other two parameters were *λ* = 0.0079 min^-1^ and *Π* = 18.30 min. All three means were close to our previous estimates based on pooled data. The rounded values of *a* = .48 and σ_S_^2^ = .00046 are used hereafter.

Thus, although asymmetrical partitioning creates in a deterministic manner a difference between new and old daughters, it is still subject to stochasticity or noise. The mean values *a* = .48 and *(1 –a)* = .52 reflect the deterministic process, and σ_S_^2^ = .00046 represents the magnitude of the stochastic component.

### Fitness advantage of stochastic and asymmetrical partitioning

Natural selection for stochastic and asymmetrical partitioning was modeled by creating a computational model for a population of bacteria. Following descriptions presented in Methods, the population was propagated forward in time with Eqs 4, 5, 7 and 8, subjected to natural selection for shorter doubling times, and monitored the resulting relative fitness. We first compared a simulation of three populations: a symmetrical population (*a* = ½ and σ_S_^2^ = 0), a stochastic population (*a* = ½ and σ_S_^2^ = .00046), and an asymmetrical population (*a* = .48 and σ_S_^2^ = .00046). The values of *Π* = 18.30 min and *λ* = 0.0095 min^-1^ were used for the analysis. The value of *λ* was set higher because previous analyses have shown that the fitness advantage of asymmetrical partitioning is negligible when *λ* is small [20]. Elevating *λ* favors asymmetrical partitioning because a bacteria that partitions symmetrically cannot survive if *λ* >1/6*Π* [20]. Thus, if *Π* = 18.30, *λ* = 0.0095 min^-1^ > 1/6*Π*.

**Fig 4:**
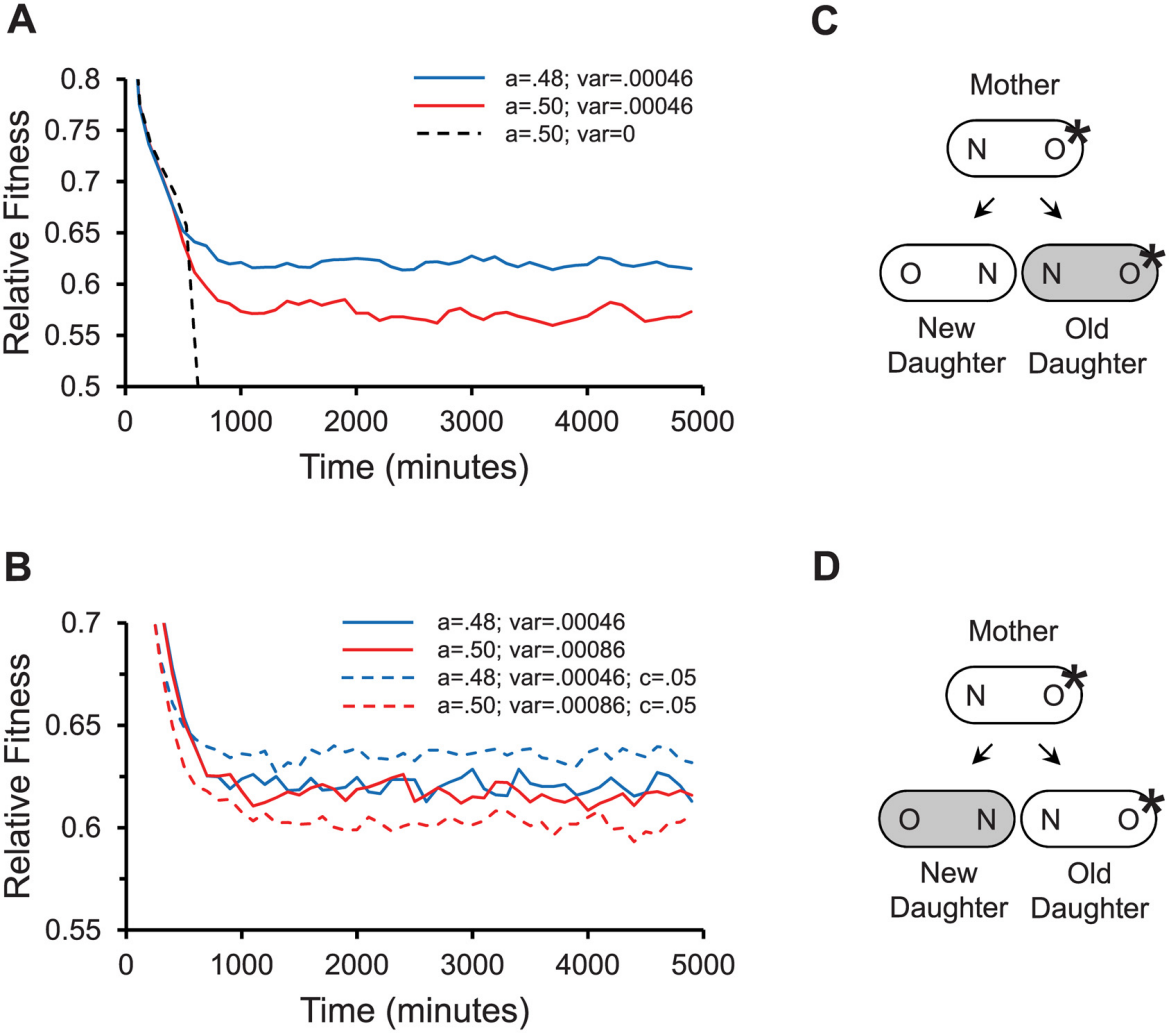
Modeling fitness for damage partitioning in bacteria. Results report relative fitness over time for populations propagated in a computer model as described (Methods). Parameter values of *λ* = .0095 min^-1^ and Π = 18.30 min were used for all simulations. A relative fitness of .5 corresponds to a severely damaged and effectively dead cell that no longer can divide. Because fitness stabilizes after about 1500 min with these parameter values, fitness values between 1500 to 5000 min were used to calculate mean fitness. (A) Relative fitness over time for asymmetrical partitioning with stochasticity (*a* = .48; var = σ_S_^2^ = .00046); symmetrical partitioning with stochasticity (*a* = ½; var = σ_S_^2^ = .00046); and symmetrical partitioning with no stochasticity (*a* = .5; var = 0). (B) Relative fitness over time for asymmetrical partitioning with stochasticity (*a* = .48; var = σ_S_^2^ = .00046; no anchored damage); symmetrical partitioning with elevated stochasticity (*a* = ½; var = σ_S_^2^ + *D* ^2^/4 = .00046 + .0004^2^/4 = .00086; no anchored damage); asymmetrical partitioning with stochasticity (*a* = .48; var = σ_S_^2^ = .00046; with anchored damage *C* = .05); symmetrical partitioning with elevated stochasticity (*a* = ½; var = σ_S_^2^ + *D* ^2^/4 = .00046 + .0004^2^/4 = .00086; with anchored damage *C* = .05). (C) Anchored damage in asymmetrically produced daughters. Because asymmetrical partitioning (gray shading) allocates movable damage to the old daughter and anchored damage (_*_) is more likely to appear first in the mother’s older pole, the difference between old and new daughters is magnified. The magnification increases the variance of damage partitioning. (D) Anchored damage in symmetrically produced daughters. If partitioning is symmetric but stochastic, 50% of the time movable damage is allocated to the old daughter as in Fig 4C. However the other 50% of the time it is as depicted here, where movable damage (gray shading) is allocated to the new daughter and anchored damage (_*_) is in the old daughter. The old and new daughters are rendered more similar and the variance of damage partitioning is reduced.

Simulations of the propagation of the three populations showed distinctly different outcomes (Fig 4A). As predicted, the symmetrical population was unable to persist with such a high *λ*; it went extinct. The stochastic population achieved an intermediate mean fitness that equilibrated around 0.571, while the asymmetrical population fared even better and had a mean fitness of 0.620. However, it is remarkable that the stochastic population was able to survive the damage, when the symmetrical population was not. Its ability to handle damage results from the fact that stochastic partitioning also introduces asymmetry. If a stochastic population partitions damage between old and new daughters with *a* = ½ and σ_S_^2^ = .00046, the distribution of damage is randomly distributed in regards to the polarity of daughters. A new daughter is just as likely to get more damage as an old daughter (Fig 3B). As a result, it makes no functional difference, from the perspective of damage partitioning, to categorize the two daughters descending from the same mother as old and new. However, it is functionally relevant to categorize them as to the one receiving either more or less damage. The distribution of the ones receiving less becomes then a folded or half-normal distribution. Because a half-normal distribution has a mean of ½—√(σ_S_^2^٠2 / 3.141593…) = .483, half of the daughters in a stochastic population are receiving on the average a small percentage of the damage from the mother (Fig 3C). Thus, stochastic partitioning is effectively asymmetric.

### Matching fitness of stochastic and asymmetrical partitioning

By being effectively asymmetric, stochastic partitioning, in the same manner as asymmetrical partitioning, provides a fitness advantage by increasing the variance of the amount of damage the daughter bacteria receive. The fitness advantage provided by the two mechanisms should match when the resulting variances are equal. This point of equality can be estimated.

Let *V*_*S*_, *V*_*New*_, and *V*_*Old*_ represent the variances of the population of daughters produced by stochastic partitioning and the populations of new and old daughters created by asymmetrical partitioning. Because the amount of damage in the new and old daughters is generated by the same process in the same mother, *V*_*New*_ = *V*_*Old*_ and the two variances are estimated by our value of σ_S_^2^ = .000456. Although we assumed earlier that *V*_*S*_ = *V*_*New*_ = *V*_*Old*_, we will now allow *V*_*S*_ to increase to determine when equality ensues. Although the variances of the three populations can be the same, their means are not. While the mean of the stochastic population is *½*, they are *(½ –D/2)* and *(½ + D/2)* in the new and old daughter populations, where *D* is the difference in the mean proportion of damage asymmetrical partitioning allocates to the old and new daughters. As we estimated the mean proportion to the new daughter to be .48 (see above), *D = (1–*.*48)–*.*48 = 0*.*04*.

Thus,

$$V_{S}=\;\sum^{\text{​}}\;f\left(i\right)\;{\left[a\left(i\right)\;–\;½\right]}^{2}$$

$$V_{New}=\;\sum^{\text{​}}\;g\left(i\right)\;{\left[x\left(i\right)\;–\;\left(½–D/2\right)\right]}^{2}$$

$$V_{Old}=\;\sum^{\text{​}}\;g\left(i\right)\;{\left[y\left(i\right)\;–\;\left(½+D/2\right)\right]}^{2}$$

where *a(i)*, *x(i)*, and *y(i)* are the *ith* amount of damage received by individuals in the stochastic, new, and old populations, and *g(i)* and *f(i)* are their frequencies. If *g(i) = f(i)*, the partitioning of damage is subject to the same level of stochasticity or noise within the three population.

The total variance created by asymmetrical partitioning results from combining the new and old daughters into a single population. The mean of this combined population is also *½*, and thus its total variance is

$$V_{Total}=\;\left\{\;\sum^{\text{​}}\;g\left(i\right)\;{\left[x\left(i\right)\;–\;½\right]}^{2}+\sum^{\text{​}}\;g\left(i\right)\;{\left[y\left(i\right)\;–\;½\right]}^{2}\right\}\;/\;2$$

The division by 2 is needed because there are two summations. By substituting and rearranging, and noting that *V*_*New*_ = *V*_*Old*_, the result simplifies to (see Methods for complete derivation)

$$V_{Total}=V_{New}+D^{2}/4$$

By using our previous estimates of *V*_*New*_ = σ_S_^2^ = .00046 and *D* = .04, *V*_*Total*_ = .00086.

Thus, we predict that if the variance generated by stochastic partitioning equals *V*_*Total*_ = .00086, a stochastic population should have the same relative fitness as an asymmetric population (Fig 3D). We tested our prediction by comparing the fitness of a stochastic population with *a* = .5 and σ_S_^2^ = .00086 and an asymmetrical population with *a* = .48 and σ_S_^2^ = .00046, while holding *Π* and *λ* at their previous values. Following our described protocols, we simulated the populations by propagating them with natural selection (Fig 4B). Supporting our estimate that the fitness of two populations should be equal when their variances differ by *D* ^*2*^ */4*, the fitness of the stochastic and asymmetric populations fluctuated around a mean of 0.617 and 0.620, respectively.

The close match between the two populations raises the question of why bacteria evolved to partition damage asymmetrically between their old and new daughters. Given that the partitioning of damage is inherently stochastic or noisy in bacteria, as evidenced by our estimate of σ_S_^2^ = .00046 for the fraction of damage allocated to only the new daughter, it follows that bacteria could have achieved equivalent fitness gains by simply evolving a higher level of stochasticity. Evolving asymmetrical partitioning may have been less costly than evolving higher stochasticity, but that raises the second question as to why asymmetrical partitioning biased the allocation of damage to the old daughter, which is the one harboring the older pole of the mother (Fig 2). An answer to both questions may be that the older pole of the mother, by virtue of its higher age, has more damage in anchored and slow-turnover macromolecules, e.g. polar mureins and flagellar motor rings [28, 29]. Mureins that form the peptidoglycan structures of cell wall in *E*. *coli* are deposited at new poles of daughter cells only at the time they are formed when the mother cell divides (Fig 2). While new mureins are constantly added to the side walls during growth of the daughter cells, the polar mureins remain inert. The presence of such anchored damage polarizes the evolution because variance is increased when non-anchored damage is partitioned to the old daughter (Fig 4C and 4D).

### Modeling effects of anchored damage

To test our prediction that some anchored damage may have triggered the evolution of asymmetrical partitioning, we modified our basic model to allow for the buildup of anchored damage in the old pole of the mother. A new parameter *C* was introduced to represent the fraction of anchored damage. Thus, a fraction *(1-C)* of non-anchored damage is still asymmetrically partitioned as before, and anchored damage accumulates at a rate *C λ* and non-anchored damage at a rate *(1 –C) λ*. The amount of anchored and non-anchored damage in a mother cell at time *t* is *v(t)* and *w(t)* respectively, and

$$v\left(t\right)\;=\;v_{0}+\;C\;\lambda \;t$$

$$w\left(t\right)\;=\;w_{0}+\;\left(1\;–\;C\right)\;\lambda \;t$$

$$k_{0}=\;v_{0}+\;w_{0}$$

where *v*_*0*_ and *w*_*0*_ are the amounts of anchored and non-anchored damage the cell receives at birth from her own mother and *k*_*0*_ is redefined to represent the total amount of damage. Because

$$\begin{array}{lll} k\left(t\right) & = & v\left(t\right)\;+\;w\left(t\right)\\ & = & k_{0}+\;\lambda \;t \end{array}$$

Eqs 1, 2, 3 and 8 in the basic model (see Methods) are still valid with anchored damage. However Eqs 4 and 5 needed to be modified. Because asymmetric partitioning gives the new daughter a fraction *a* of the non-anchored damage and none of the anchored damage

$$\begin{array}{lll} k_{1} & = & w\left(T_{0}\right)\;a\\ k_{2} & = & w\left(T_{0}\right)\left(1\;–\;a\right)\;+\;v\left(T_{0}\right) \end{array}$$

With these and no additional modifications, Eqs 6 and 7 and the basic model could be used to describe the effect of anchored damage on evolution by natural selection in a bacterial population. Stochasticity was added as before by sampling *a* from a Gaussian distribution.

The effect of anchored damage was first investigated by comparing the fitness of stochastic (*a* = .5 and σ_S_^2^ = .00086) and asymmetric (*a* = .48 and σ_S_^2^ = .00046) bacterial populations with *C* = .05. While mean fitness varied previously around similar values of .617 and .620 in the control stochastic and asymmetric populations (Fig 4B; no anchor), it decreased to .602 in the stochastic population and increased to .636 in the asymmetric population in the presence of anchored damage (Fig 4B; *C* = .05). Thus, anchored damage has a strong effect on polarizing evolution to favor the asymmetrical partitioning of damage to the old daughter.

**Fig 5:**
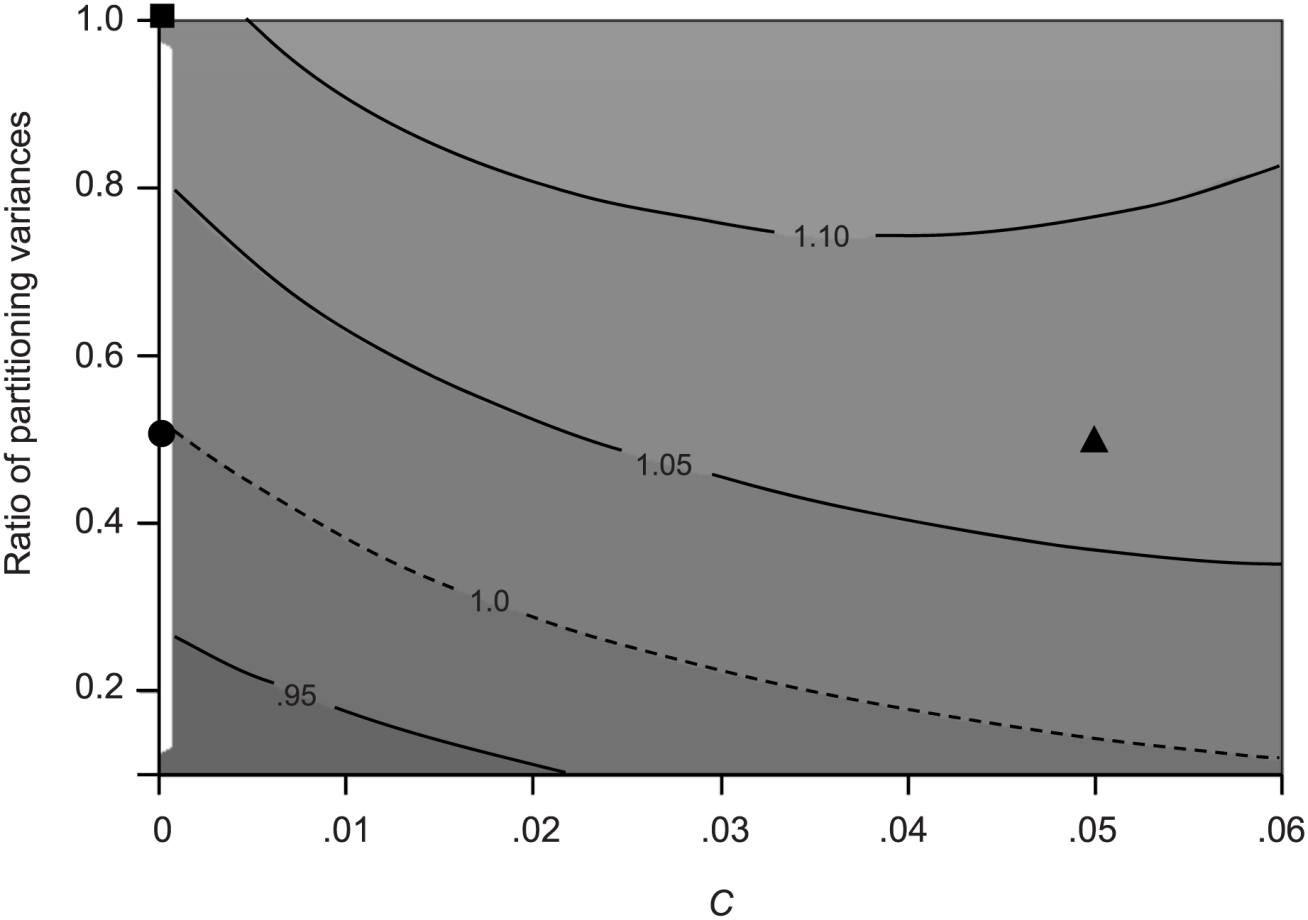
Fitness landscape for damage partitioning with anchored damage. Landscape compares asymmetric and stochastic bacteria (*a* = .48; var = .00046) with symmetric and stochastic bacteria (*a* = ½; variance explored over a range of .0046 to .00046). The partitioning variance of asymmetric bacteria was held constant because this value was the estimate obtained from experimental data in *E*. *coli*. All reported ratios are for values of asymmetric bacteria divided by values of symmetric bacteria. Contour lines represent the fitness ratio of mean relative fitness determined from simulated populations after values stabilized (see Fig 4). Parameter values of *λ* = .0095 min^-1^ and Π = 18.30 min were used for all simulations. The x-axis represents values of the fraction *C* of anchored damage. The y-axis represents the ratio of the partitioning variance. Region above contour line 1.0 represent *C* and variance ratio values for which asymmetric bacteria have higher fitness. The points (■, ●, and ▲) on the surface denote fitness ratios of populations previously presented, respectively, in Fig 3A (variance of symmetric bacteria = .00046; no anchor), Fig 3B (variance of symmetric bacteria = .00086; no anchor), and Fig 3B (variance of symmetric bacteria = .00086; *C* = .05).

To examine more broadly the effects of *C* and σ_S_^2^ on evolution, we explored how the fitness values of asymmetric and stochastic populations responded to changes in the two parameters (Fig 5). As we had found before, when there was no anchored damage (*C* = 0) the fitness ratio of the two populations was 1.0 when the variance (σ_S_^2^) ratio equaled .00046 / .00086 = .53, all ratios reported as asymmetric over stochastic. However, as *C* was initially increased from zero, the variance ratio needed to be decreased for the fitness ratio to remain equal to 1.0. In other words, increasing *C* harmed the stochastic population, which then needed to increase its variance to compensate. Anchored damage is harmful because it reduces variance in the stochastic population by opposing the effects of stochastic partitioning (see Fig 4D). The fitness advantage provided by anchored damage to the asymmetric population did diminish as *C* was increased further, as demonstrated by the leveling of the fitness isoclines. The reason is because as *C* increases, the fraction of anchored damage becomes sufficiently large to override the effects of asymmetrical partitioning. In other words, the variance in both asymmetric and stochastic populations becomes largely created by anchored damage, which is always polarized. Nonetheless, the effect of small values of *C* shows clearly that small amounts of anchored damage are needed to promote the evolution of asymmetrical partitioning.

## Discussion

Evolution by natural selection requires phenotypic variation in fitness, and it proceeds more rapidly, other things being equal, with added variation. The added variance increases the efficiency of natural selection. In evolutionary biology, mutations and sexual reproduction (recombination and reassortment) have been considered to be the major sources of variation. The production of asymmetrical daughters by seemingly symmetrical *E*. *coli* [9] adds a novel, and potentially important, source of variation. Unlike mutations and sexual reproduction, asymmetrical partitioning does not cause genetic or DNA changes. However, the partitioning of damage is heritable because mothers with more damage produce daughters with higher mean levels of damage. It is equivalent to a maternal effect that results from the provisioning of yolk, other gene products, mRNA’s, or nutrition by a mother to her offspring [1]. The only difference is that the asymmetry increases the variance within the progeny of daughters. The allocation of more damage to the older daughter has been used as model for the evolution of aging [8, 9]. Over time a lineage of old daughters can acquire a larger load of damage and experience functional deterioration. Thus, aging is one of the evolutionary costs that cells pay for phenotypic variation.

Although our results demonstrate how asymmetric partitioning is evolutionarily advantageous, they also show how stochastic variance created by random noise can be similarly beneficial. Because a population that is stochastic and symmetrical is effectively asymmetric, it also creates daughters that have different levels of damage (Figs 3, 4 and 5). Had asymmetric partitioning not evolved, stochastic partitioning could have been one of the most compelling examples of beneficial stochasticity. Demonstrating the advantage of stochasticity is difficult because models invoking stochasticity generally assume a bet-hedging strategy in a changing environment. For many biological processes, the probability and magnitude of the fitness payoffs in the new environment are not readily determined. For stochastic partitioning, the effect of random variation on the doubling time of old and new daughters is easily translated into fitness and natural selection.

Our results can be used to estimate the stochastic and deterministic contributions to the variance of damage partitioning. If σ_S_^2^ = .00046 is the stochastic variance of damage partitioning in separate populations of new and old daughters, then the total variance in the entire population is σ_S_^2^ + *D* ^2^/4 = .00046 + .0004 = .00086. Because the difference *D* between the means of the new and old daughter populations is deterministically caused by the biology and genetics of *E*. *coli*, *D* ^2^/4 = .0004 is deterministic variance. Thus, nearly ½ of the total variance of damage partitioning is deterministic, or (*D* ^2^/4) / (σ_S_^2^ + *D* ^2^/4) = 47%. The remaining σ_S_^2^ / (σ_S_^2^ + *D* ^2^/4) = 53% is due to stochasticity.

Because asymmetrical partitioning is the extant phenotype of *E*. *coli*, one could postulate that the ancestral state was 100% stochastic. Stochasticity may have been critically needed to elevate fitness early in evolution, perhaps when the first proto-cells began to evolve. The irony is that the greater the advantage of stochasticity, the stronger natural selection would have favored supplanting it with a deterministic adaptation such as asymmetric partitioning. Repairing damage is another solution [21, 30, 31], and we expect that to occur and dampen the selection. However, given that asymmetric partitioning is apparent over a generation [9, 12, 20, 25, 26], a significant fraction of damage must be either non-repairable or not easily repaired over that time scale. Because asymmetrical partitioning evolved to allocate more damage to the older daughter, we postulated that the bias could have been triggered by the presence of immovable damage that was anchored to the older pole of the mother cell. Our computational model showed that the conditions for its evolution were favorable and required a small proportion of anchored damage. We postulate that anchored damage constitutes only a small proportion because if it could grow to be 100% of a cell’s damage, an old daughter lineage should eventually die from the buildup. The empirical observation is instead that the lineage does not die and damage levels converge to equilibrium levels [20, 25]. Thus, a significant proportion of damage in the old daughter cannot be anchored and it is redistributed, albeit asymmetrically, between the old and new daughters.

If our reconstruction of the evolution of asymmetric partitioning is correct, the supplanting of stochastic process by a deterministic one could constitute a microbial example of Waddington’s genetic assimilation. The main difference is that it was a phenotypic trait, and not its variance, that was assimilated in Waddington’s crossveinless example. However, variance is as good of a trait as any other quantifiable phenotype, and variance was under selection in our model. Moreover, if an increase in the variance of the activation factor, as we proposed in Fig 1C, accounts for the assimilation of crossveinless, the evolution of asymmetric partitioning and crossveinless becomes much more comparable. In both cases, the evolution requires the emergence of a deterministic mechanism to generate variation.

## Methods

### The basic model

The model assumes that the amount of damage in a mother cell at any time *t* is

$$k\left(t\right)\;=\;k_{0}+\;\lambda \;t$$ (1)

where *k*_*0*_ is the amount of damage the cell receives at birth from her own mother and *λ* is the rate at which new damage accumulates. The mother cell divides when it has built up an intracellular product *P* to a threshold quantity *Π*. Assuming that damage hinders function linearly, *P* accumulates at a rate

$$\begin{array}{lll} dP/dt & = & 1\;–\;k\left(t\right)\\ & = & 1\;–\;k_{0}–\;\lambda \;t \end{array}$$

$$P\left(t\right)\;\;\;\;=\;\left(1\;–\;k_{0}\right)\;t\;–\;\left(\lambda \;/\;2\right)\;t^{2}$$

by integration. When *P(t) = Π*, the mother cell divides. Denoting that time point *t* = *T*_*0*_ as her doubling time, *P(T*_*0*_ *) = Π* and

$$\Pi \;=\;\left(1\;–\;k_{0}\right)\;T_{0}–\;\left(\lambda \;/\;2\right)\;T_{0}{}^{2}$$ (2)

The integration constant *P(*0*)* is set to zero because a new pool of the product *P* is assumed to be built *de novo* for every cell division. At the time of division, the mother cell partitions her damage *k(T*_*0*_) to her two daughters and

$$k(T_{0})=\;k_{0}+\;\lambda T_{0}$$ (3)

*k(T*_*0*_) is partitioned asymmetrically to the cell’s daughters in the proportions *a* and *(1 –a)*. Thus, the daughters receive

$$k_{1}=\;(k_{0}+\;\lambda T_{0})a$$ (4)

$$k_{2}=\;(k_{0}+\;\lambda T_{0})(1\;–\;a)$$ (5)

where 0 ≤ *a* ≤ ½ and the subscripts 1 and 2 denote the new and old daughters. When each daughter in turn becomes a mother, Eq 2 can be resubscripted to annotate the daughters or

$$\Pi \;=\;\left(1\;–\;k_{i}\right)\;T_{i}–\;\left(\lambda \;/\;2\right)\;T_{i}{}^{2}$$ (6)

$$T_{i}=\;\left\{\left(1\;–\;k_{i}\right)\;–\;\surd \left(\;{\left(1\;–\;k_{i}\right)}^{2}–\;2\;\Pi \;\lambda \;\right)\right\}\;/\;\lambda$$ (7)

by the quadratic formula and *i = 1* or *2*.

Thus, given *T*_*0*_ for a mother cell, *T*_*i*_ of her two daughters can be determined. *k*_*0*_ in Eq 3 is obtained by rearranging Eq 2 as

$$k_{0}=\;1\;–\;\left(\lambda \;/\;2\right)\;T_{0}–\;\Pi \;/\;T_{0}$$ (8)

### Propagating populations with selection in the computational model

After values of the parameters *a*, σ_S_^2^, *λ*, and *Π* were chosen to represent stochastic and asymmetrical partitioning, the starting bacterial population of 1000 individuals was established with no initial damage (*k*_*0*_ = 0). With Eq 8, *T*_*0*_ was determined and used in conjunction with Eqs 4, 5 and 7 to predict the *T*_*1*_ and *T*_*2*_ values for the population of cells the next generation. After reproduction the population was randomly culled to reduce it to 1000 individuals. The process was then iterated forward in time by letting the surviving cells reproduce, which was accomplished by letting their *k*_*1*_, *T*_*1*_, *k*_*2*_ and *T*_*2*_ values serve as *k*_*0*_ and *T*_*0*_ for the next iteration, and so forth until the mean doubling time of the population remained stable for a sufficiently long time (about 5000 minutes; e.g. Fig 4A and 4B).

Natural selection and evolution were imposed spontaneously in the model by scaling time to minutes instead of generations and allowing cells to reproduce only after an increment equal to their doubling time had passed. Cells with shorter doubling times divided more often, and hence were more fit and favored by natural selection.

### Calculating relative fitness

Because doubling times are inversely proportional to fitness, *T*_*0*_, *T*_*1*_ and *T*_*2*_ need to be converted to relative fitness. The proper conversion is to compare doubling times relative to *Π* [20], which is the doubling time of the most fit and damage free cell (see above). The number of damage free cells increases by a factor of 2 in a time interval *Π*. A cell with a doubling time of *T*_*i*_ increases by a factor of 2^*Π/Ti*^ over the same interval. Thus,

$$\begin{array}{ccc} W & = & 2^{\Pi /Ti}/\;2\\ & = & 2^{\left(\Pi /Ti\right)}{}^{–\;1} \end{array}$$

A fitness of 1 indicates that a cell has the shortest possible doubling time of the fittest cell. A cell so overloaded with damage that it cannot divide has an infinitely long doubling time, in which case its relative fitness has the lowest possible fitness value of 0.5. The latter results because the cell persists in the population, but its presence as one cell accounts for only 0.5 of the two daughters made by the fittest cell. Most cells have doubling times and fitness values that fall between these extremes. A population with a mean fitness value of 0.5 goes extinct because it is unable to reproduce.

Mean fitness for populations were determined after doubling times were observed to reach a stable range of values. Noting that all our populations stabilized after about 1500 minutes, mean fitness was determined within the window of 1500 to 5000 min (Fig 4A and 4B).

$$SolvingforV_{Total}=V_{New}+D^{2}/4$$

Equation and notation as described in text.

$$V_{Total}=\;\left\{\;\sum^{\text{​}}\;g\left(i\right)\;{\left[x\left(i\right)\;–\;½\right]}^{2}+\sum^{\text{​}}\;g\left(i\right)\;{\left[y\left(i\right)\;–\;½\right]}^{2}\right\}\;/\;2$$

Adding *(D/2 –D/2)* to the inside of the summations, rearranging, and combining similar terms

$$\begin{array}{lll} 2\;V_{Total} & = & \sum \;g\left(i\right){\left[x\left(i\right)\;–\;½\;+\;\left(D/2–D/2\right)\right]}^{2}+\sum \;g\left(i\right){\left[y\left(i\right)\;–\;½\;+\;\left(D/2\;–D/2\right)\right]}^{2}\\ & = & \sum \;g\left(i\right){\left[x\left(i\right)\;–\;\left(½–D/2\right)\;–\;D/2\right]}^{2}+\sum \;g\left(i\right){\left[y\left(i\right)\;–\;\left(½+D/2\right)\;+\;D/2\right]}^{2}\\ & = & \sum \;g\left(i\right)\left[x{\left(i\right)}^{2}–2x\left(i\right)\left(½–D/2\right)+{\left(½–D/2\right)}^{2}–\;D\left(½–D/2\right)\;+\;Dx\left(i\right)\;+\;D^{2}/4\right]\\ & & \;+\;\sum \;g\left(i\right)\left[y{\left(i\right)}^{2}–2y\left(i\right)\left(½+D/2\right)+{\left(½+D/2\right)}^{2}–\;D\left(½–D/2\right)\;+\;Dy\left(i\right)\;+\;D^{2}/4\right]\\ & = & \sum \;g\left(i\right)\left[x{\left(i\right)}^{2}–2x\left(i\right)\left(\;–D/2\right)+{\left(½–D/2\right)}^{2}\right]\\ & & –\;D\left(½–D/2\right)\;+\;D\sum \;g\left(i\;\right)x\left(i\right)\;+\;D^{2}/4\\ & & +\;\sum \;g\left(i\right)\left[y\left(i\right)\;–2y\left(i\right)\left(½+D/2\right)+{\left(½+D/2\right)}^{2}\right]\\ & & –\;D\left(½–D/2\right)\;+\;D\sum g\left(i\;\right)y\left(i\right)\;+\;D^{2}/4 \end{array}$$

Noting that *∑ g(i) x(i) = (½ –D/2)* and *∑ g(i) y(i) = (½+D/2)* are the means of the new and old daughter populations, *∑ g(i) = 1*, *∑ g(i)k = k* if *k* is a constant, and *V*_*New*_ *= V*_*Old*_,

$$\begin{array}{lll} 2\;V_{Total} & = & \sum^{\text{​}}\;g\left(i\right){\left[x\left(i\right)\;–\;\left(½–D/2\right)\right]}^{2}–\;D\left(½–D/2\right)\;+\;D\left(½–D/2\right)\;+\;D^{2}/4\\ & & +\;\sum^{\text{​}}\;g\left(i\right){\left[y\left(i\right)–\left(½+D/2\right)\right]}^{2}–\;D\left(½+D/2\right)\;+\;D\left(½+D/2\right)\;+\;D^{2}/4\\ & = & V_{New}+\;V_{Old}+2\;D^{2}/4 \end{array}$$

$$\;V_{Total}=V_{New}+D^{2}/4$$
